# 18*β*-glycyrrhetyl-3-*O*-sulfate would be a causative agent of licorice-induced pseudoaldosteronism

**DOI:** 10.1038/s41598-018-38182-2

**Published:** 2019-02-07

**Authors:** Kan’ichiro Ishiuchi, Osamu Morinaga, Takeshi Ohkita, Chuanting Tian, Asuka Hirasawa, Miaki Mitamura, Yasuhito Maki, Tsubasa Kondo, Tomoya Yasujima, Hiroaki Yuasa, Kiyoshi Minamizawa, Takao Namiki, Toshiaki Makino

**Affiliations:** 1 Department of Pharmacognosy, Graduate School of Pharmaceutical Sciences, Nagoya City University, 3-1 Tanabe-Dori, Mizuho-ku, Nagoya Japan; 2 Department of Natural Medicines, Daiichi University of Pharmacy, 22-1 Tamagawamachi, Minami-ku, Fukuoka Japan; 3 Department of Biopharmaceutics, Graduate School of Pharmaceutical Sciences, Nagoya City University, 3-1 Tanabe-Dori, Mizuho-ku, Nagoya Japan; 4 Department of Oriental Medicine, Kameda Medical Center, 929 Higashi-cho, Kamogawa, Japan; 5 Department of Japanese-Oriental (Kampo) Medicine, Chiba University Hospital, 1-8-1 Inohana, Chuo-ku, Chiba-shi, Chiba Japan

## Abstract

Licorice-induced pseudoaldosteronism is a common adverse effect in traditional Japanese Kampo medicine, and 3-monoglucuronyl glycyrrhetinic acid (3MGA) was considered as a causative agent of it. Previously, we found 22*α*-hydroxy-18*β*-glycyrrhetyl-3-*O*-sulfate-30-glucuronide (**1**), one of the metabolites of glycyrrhizin (GL) in the urine of Eisai hyperbilirubinuria rats (EHBRs) treated with glycyrrhetinic acid (GA), and suggested that it is also a possible causative agent of pseudoaldosteronism. The discovery of **1** also suggested that there might be other metabolites of GA as causal candidates. In this study, we found 22*α*-hydroxy-18*β*-glycyrrhetyl-3-*O*-sulfate (**2**) and 18*β*-glycyrrhetyl-3-*O*-sulfate (**3**) in EHBRs’ urine. **2** and **3** more strongly inhibited rat type 2 11*β*-hydroxysteroid dehydrogenase than **1** did *in vitro*. When EHBRs were orally treated with GA, GA and **1**–**3** in plasma and **1**–**3** in urine were detected; the levels of 3MGA were quite low. **2** and **3** were shown to be the substrates of organic anion transporter (OAT) 1 and OAT3. In the plasma of a patient suffering from pseudoaldosteronism with rhabdomyolysis due to licorice, we found 8.6 µM of **3**, 1.3 µM of GA, and 87 nM of **2**, but **1**, GL, and 3MGA were not detected. These findings suggest that 18*β*-glycyrrhetyl-3-*O*-sulfate (**3**) is an alternative causative agent of pseudoaldosteronism, rather than 3MGA and **1**.

## Introduction

It is well known that excessive intake of licorice induces pseudoaldosteronism, which is characterized by peripheral edema, hypokalemia, and hypertension^1^. Licorice, the roots of *Glycyrrhiza glabra* or *G*. *ularensis*, is used as a crude drug in traditional Japanese Kampo and traditional Chinese medicine. It is actually utilized in more than 70% of the Kampo formulas approved by the Japanese Medicinal Regulatory Agency, the Ministry of Health Labour and Welfare of Japan^2^. Pseudoaldosteronism is a common adverse effect of Kampo medicine. Since this condition is sometimes life-threatening^3^, its early detection is critical to prevent disease aggravation. Although the frequency with which pseudoaldosteronism caused by Kampo medicines arises depends to some extent on the dosage and duration of licorice treatment^4^, its onset exhibits large individual differences and it is generally unpredictable.

**Figure 1:**
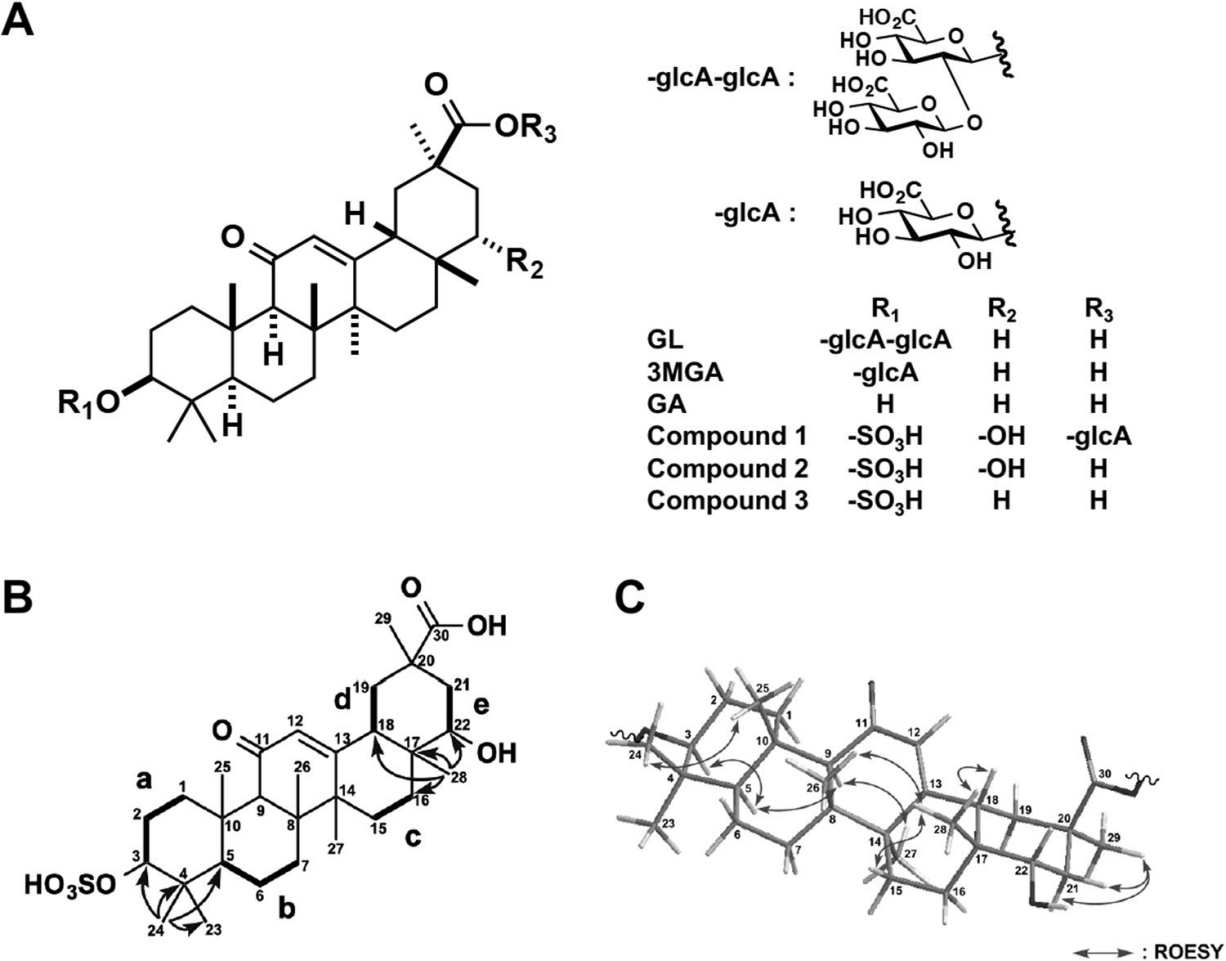
Chemical structures of glycyrrhizin (GL) and its metabolites. Chemical structures of GL metabolites in this study (**A**). Selected 2D NMR correlations for compound **2** (**B**). Selected ROESY correlations for compound **2** (**C**).

The causative agents of licorice-induced pseudoaldosteronism have been studied for more than 30 years. Glycyrrhizin (GL, Fig. 1A) is the main active ingredient of licorice, and it is used as an anti-inflammatory agent^5^ and a natural sweetener^6^ in its pure form. When licorice is taken orally, GL cannot be absorbed by the intestine in its original form and is absorbed as glycyrrhetinic acid (GA, Fig. 1A) only after it is hydrolyzed by an enzyme of the intestinal bacterial flora^7^. Monder *et al*. evaluated the inhibitory effects of GL and GA on type 2 11*β*-hydroxysteroid dehydrogenase (11*β*-HSD2) using a rat liver homogenate and found that the titer of GA was approximately 200-fold higher than that of GL. Since human consumption of licorice led to plasma concentrations of GA that were higher than those of GL, it is suggested that the causative agent of licorice-induced pseudoaldosteronism GA^8^.

Kanaoka *et al*. found 3-monoglucuronyl-glycyrrhetinic acid (3MGA, Fig. 1A) in the serum of a patient with GL-induced pseudoaldosteronism^9^. Moreover, among patients who took GL, Kato *et al*. found 3MGA in the blood samples of 10 patients who exhibited pseudoaldosteronism, but not in the blood samples of 11 patients without this condition, suggesting that 3MGA, not GA, is the causative agent of licorice-induced pseudoaldosteronism^10^. In our previous studies, we found that 3MGA did not appear in the plasma or urine of Sprague-Dawley (SD) rats treated with GA, but did appear in rats with liver fibrosis and in multidrug resistance-associated protein (Mrp) 2-deficient Eisai hyperbilirubinuria rats (EHBRs). This suggested that the circulating GA was further metabolized to 3MGA in the liver by UDP-glucuronyltransferase and then excreted in the bile *via* Mrp2 into the intestine^11,12^. Intestinal bacteria hydrolyzed the biliary-excreted 3MGA back into GA, which was then reabsorbed into the bloodstream^11,13^.

Kato *et al*. could not explain the mechanisms by which only 3MGA might cause pseudoaldosteronism, since GA and 3MGA exhibited similar inhibitory effects on 11*β*-HSD2 using a rat kidney microsome fraction *in vitro*, which was consistent with the blood concentrations of GA in patients who took GL and exhibited pseudoaldosteronism^10^. Ohtake *et al*. reported that only 3MGA caused hypokalemia when GA or 3MGA was injected intravenously (*i*.*v*.) into guinea pigs, suggesting that the disposition of 3MGA might play roles in the pathogenesis of pseudoaldosteronism^14^. It was revealed that only 3MGA was a substrate of organic anion transporter (OAT) 1 and OAT3, capable of being transported into tubular epithelial cells^15^. Since 11*β*-HSD2 is located in the microsomes of tubular epithelial cells^16^, GA may not be able to inhibit 11*β*-HSD2 *in vivo*. Rather, 3MGA may inhibit 11*β*-HSD2, leading to an increase in cortisol level and the subsequent elevation of sodium retention and potassium excretion, causing pseudoaldosteronism^10,17^.

From these results, we developed an anti-3MGA-monoclonal antibody (mAb) and an enzyme-linked immunosorbent assay (ELISA) to easily detect 3MGA in the blood or urine of patients who took licorice, in order to prevent pseudoaldosteronism by predicting the individual body constitution causing this condition without using expensive instruments such as LC-MS/MS^18^. However, 3MGA detected by ELISA in the plasma and urine of EHBRs treated with GA was observed at concentrations much higher than those analyzed using LC-MS/MS, suggesting that the anti-3MGA-mAb cross-reacted with other metabolites of GA^18^. Eastern blot analysis using anti-3MGA-mAb with the urine from EHBRs treated with GA revealed another metabolite, 22*α*-hydroxy-18*β*-glycyrrhetyl-3-*O*-sulfate-30-glucuronide (**1**, Fig. 1A) as a new natural product^18^. Although the inhibitory titer of **1** on 11*β*-HSD2 was lower than that of 3MGA, its plasma concentration in EHBRs treated with GA was higher, making it a candidate for the causative agent of licorice-induced pseudoaldosteronism^18^.

The discovery of **1** suggested that there might be other metabolites of GA as the causal candidates for licorice-induced pseudoaldosteronism. **1** has a sulfate group at C-3, a hydroxyl group at C-22, and a glucuronic acid group at C-30. It is considered to be biosynthesized by three-step metabolic reactions *via* sulfotransferase, cytochrome P450 (CYP), and glucuronyltransferase. Therefore, it is predicted that there are other metabolites of GA that are biosynthesized by one- or two-step metabolic reactions among these three-step reactions.

In the present study, we isolated two additional metabolites of GL from the urine of EHBRs orally treated with GA and identified their chemical structures. Furthermore, we analyzed the concentrations of these GL metabolites in the plasma of the patients reported to have had licorice-induced pseudoaldosteronism with rhabdomyolysis.

## Results

### Isolation and structural elucidation of compounds 2 and 3 from EHBR urine

Female EHBRs were administered 1 mg/ml GA *via* drinking water for 3 months, and their urine was collected and pooled. From 1 liter of this urine, we isolated **2** (1.9 mg) as a new GA metabolite, and **3** (0.4 mg) that was first isolated from the bile of rats treated *i*.*v*. with GA by Jing *et al*.^19^ (Fig. 1A).

Compound **2** [[α]_D_^21^ +47 (*c* 1.0, MeOH)] exhibited a deprotonated molecule at *m/z* 565 (M-H)^−^ in the ESIMS, and the molecular formula, C_30_H_46_O_8_S, was established by HRESIMS [*m/z* 565.2835, (M-H)^−^, *Δ* + 0.0 mmu]. The ^1^H and ^13^C NMR and the HSQC spectra of **2** were similar to those of **3**, except for four signals (*δ*_C_ 76.6; *δ*_H_ 3.39, *δ*_C_ 39.6; *δ*_H_ 2.13 and 1.47, *δ*_C_ 25.8; *δ*_H_ 0.95, and *δ*_C_ 20.3; *δ*_H_ 1.81 and 1.50) in **2**. The appearance of the signals *δ*_H_ 3.39 and *δ*_C_ 76.6 and the molecular formula of **2** implied that it was a hydroxylated compound of **3**.

The planar structure of compound **2** was elucidated by analysis of 2D NMR data including the ^1^H-^1^H COSY, HSQC, and HMBC spectra in CD_3_OD. Five structural units **a** (C-1–C-3), **b** (C-5–C-7), **c** (C-15–C-17), **d** (C-18–C-19), and **e** (C-21–C-22) were disclosed by the ^1^H-^1^H COSY spectrum of **1** (Fig. 1B). The analyzes of HMBC spectra, especially including a key HMBC correlation for H_3_-28 (*δ*_H_ 0.95) to C-22 (*δ*_C_ 76.6) (Fig. 1B and Table 1), revealed that the planar structure of **2** was the same as that of an oxygenated **3** at C-22. The comparison between the chemical shifts of C-3 (*δ*_H_ 3.96 and *δ*_C_ 87.2) and C-22 (*δ*_H_ 3.39 and *δ*_C_ 76.6) of **2** suggested that a sulfate group and a hydroxy group were connected at C-3 and C-22, respectively. Thus, the planar structure of **2** was elucidated as shown in Fig. 1B.

**Table 1:** ^1^H and ^13^C NMR Data (CD_3_OD) of compound **2**.

Position	*δ*_H_^a^	*δ*_C_^b^	HMBC
1a	2.73 (1H, brd 14.0 Hz)	40.0	9, 25
1b	1.06 (1H, nd^c^)		
2a	2.07 (1H, m)	25.2	
2b	1.80 (1H, nd^c^)		
3	3.96 (1H, dd 12.0, 4.5Hz)	87.2	23. 24
4		39.9	23, 24
5	0.86 (1H, nd^c^)	56.6	7b, 23, 24, 25
6a	1.65 (1H, brd 13.0 Hz)	18.6	
6b	1.50 (1H, nd^c^)		
7a	1.77 (1H, nd^c^)	33.7	26
7b	1.46 (1H, nd^c^)		
8		46.8	9, 26, 27
9	2.49 (1H, s)	63.1	12, 25, 26
10		38.2	9, 25
11		202.6	9
12	5.57 (1H, s)	129.0	
13		171.5	15b, 27
14		45.2	9, 12, 15b, 26, 27
15a	1.81 (1H, nd^c^)	27.2	27
15b	1.29 (1H, nd^c^)		
16a	1.81 (1H, nd^c^)	20.3	28
16b	1.50 (1H, nd^c^)		
17		38.5	28
18	2.16 (1H, nd^c^)	49.8	12, 16b, 19b, 28
19a	1.82 (1H, nd^c^)	41.8	29
19b	1.77 (1H, nd^c^)		
20		44.8	29
21a	2.13 (1H, nd^c^)	39.6	29
21b	1.47 (1H, nd^c^)		
22	3.39 (1H, dd 12.5, 4.5 Hz)	76.6	21b, 28
23	1.07 (3H, s)	28.7	24
24	0.86 (3H, s)	16.9	
25	1.16 (3H, s)	17.0	9
26	1.15 (3H, s)	19.2	9
27	1.45 (3H, s)	23.9	15b
28	0.95 (3H, s)	25.8	
29	1.21 (3H, s)	28.7	
30		180.0	19b, 21b, 29

^a^500MHz. ^b^125MHz. ^c^nd: *J*-values were not determined because of overlapping with other signals.

The relative stereochemistry of **2** was deduced from ROESY data (Fig. 1C). ROESY correlations for H-3/H-5, H_3_-24/H_3_-25, H-9/H-5 and H_3_-27, H_3_-28/H-15a, H_3_-18, and H_3_-26, and H_3_-29/H-21a and H-21b revealed that the stereochemistry of **2** was the same as that of 18*β*-GA except for C-22. The α-orientation of the hydroxy group at C-22 was revealed by ^3^*J*_H-22/H2-21_ values (12.5 and 4.5 Hz). The relative stereochemistry of **2** was elucidated as shown in Fig. 1C. The CD spectrum of **2** showed a positive Cotton effect at 229 nm, similar to that of GA. Thus, the absolute configuration of **2** was established as 3*S*, 5*R*, 8*R*, 9*R*, 10*S*, 14*S*, 17*R*, 18*S*, 20*R*, and 22*S*.

### Inhibitory effects of compound 2 and 3 on rat 11*β*-HSD2

**Figure 2:**
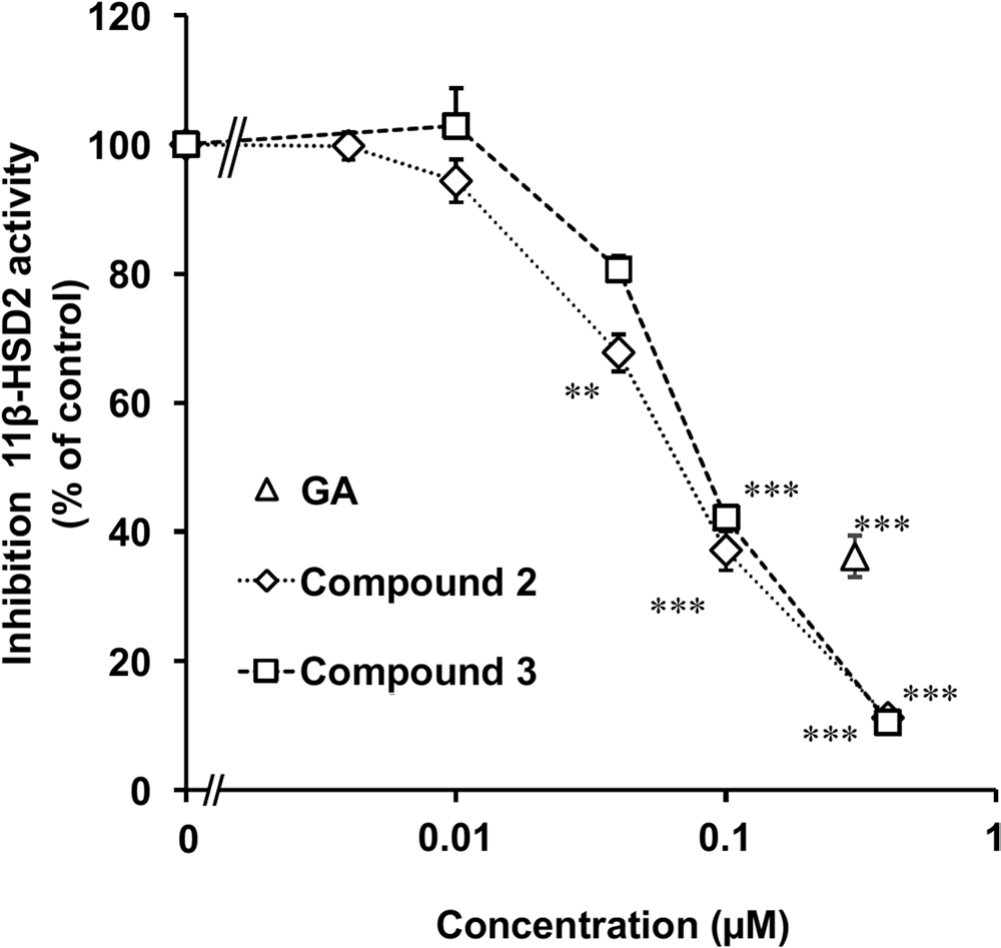
Inhibitory effects of **2** and **3** on 11*β*-HSD2 using rat kidney microsome. [^3^H] cortisone and each compound were mixed with the rat kidney microsome fraction, and incubated at 37 °C for 30 min. Then, the amount of [^3^H] cortisol was measured. Data are expressed as mean ± S.E. (*n* = 4) of the percentage relative to the amount of [^3^H] cortisol in the mixture without samples. **P < 0.01 and ***P < 0.001 compared with the groups without the samples by Dunnett’s multiple *t*-test for compounds 2 and 3, and by Student’s *t*-test for GA.

Compounds **2** and **3** inhibited 11*β*-HSD2 in a rat kidney microsome fraction in a concentrations-dependent manner (Fig. 2). Their half-maximum inhibitory concentration (IC_50_) values were 0.11 and 0.10 µM, respectively, and the inhibitory titers were slightly higher than that of GA.

### Pharmacokinetic profiles of GA and its metabolites in SD rats and EHBRs

**Figure 3:**
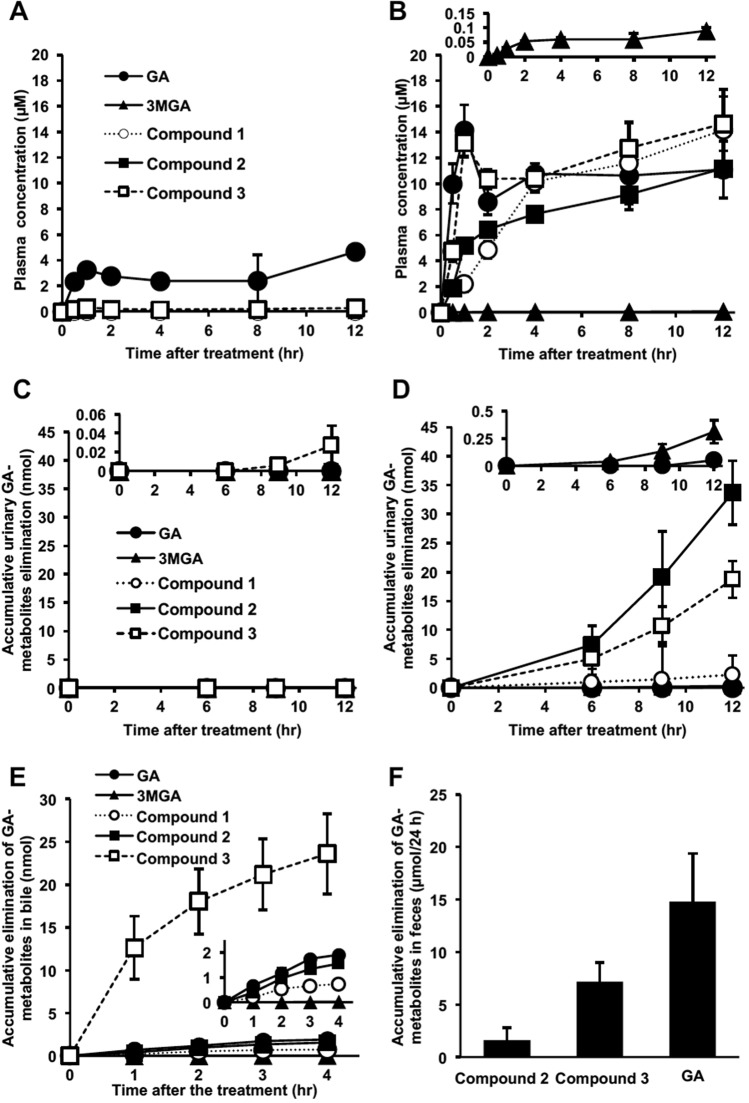
Pharmacokinetic profiles of GL metabolites in SD rats (**A**,**C**,**E**,**F**) and EHBRs (**B**,**D**) after the administration of GA. GA (200 mg/kg) was administered orally to anesthetized SD rats or EHBRs, and plasma and urine were collected for 12 h (**A**–**D**). GA (0.2 mg/kg) was intravenously injected into anesthetized SD rats in which the biliary tract was cannulated. Then, bile samples were collected for 4 h (**E**). GA (0.2 mg/kg) was intravenously injected into conscious SD rats, and the feces was collected for 24 h. 3MGA and **1** were below detectable levels in the feces (**F**). Since the determined lavels of 3MGA for B and D, **3** for C, GA for D, and all compounds except for **3** for E were relatively low; their magnified versions of their graphs are shown at the top or as an inside of each graph. The concentrations of GA metabolites were measured by LC-MS/MS, and data are plotted as mean ± S.E. (*n* = 4 for **A–D**,**F**; *n* = 3 for **E**).

We successively collected plasma and urine samples from both female SD rats and EHBRs orally administered GA (0.20 g/kg) and measured the concentrations of GA, 3MGA, and **1–3** by LC-MS/MS. Figure 3A,C show the plasma concentration profiles and urinary elimination of GA and its metabolites in SD rats, respectively; Fig. 3B,D show those in EHBRs. In SD rats, GA was appeared in the plasma at 30 min and peaked once 1 hr after the oral treatment. Then, the profile of the plasma GA concentration exhibited a biphasic curve for 12 h. The concentration of GA in the plasma was 4.7 µM at 12 h. The only other metabolite to appear in plasma was **3**, which was present at a concentration of 0.3 µM at 12 h. In the urine of SD rats, 0.03 nmol **3** was detected in the accumulated urine collected 12 h after the oral treatment, although GA, 3MGA, **1**, and **2** were below detectable levels.

In the plasma of EHBRs treated with GA, the maximum concentration of GA occured 1 h after the treatment. The concentration of GA was decreased at 2 h and increased again at 4 h, after which it was maintained for 12 h. Although the concentration of **3** at 30 min was approximately half that observed for GA, a plasma concentration profile similar to that for GA appeared after that. **2** and **1** appeared in the plasma and their concentrations gradually increased for 12 h. The concentrations of GA and **1**–**3** at 12 h after treatment were at approximately similar and all were more than 100-fold greater than that of 3MGA (0.090 µM). In the urine of EHBRs treated with GA, 3MGA and **1**–**3** were gradually eliminated, with the levels of **1**, **2**, and **3** in the accumulated urine for 12 h being 8-, 110-, and 62-fold of that of 3MGA (0.31 nmol), respectively. GA was detected in the urine at low levels (0.05 nmol).

GA, 3MGA, and compounds **1**–**3** were gradually excreted into the bile in SD rats injected *i*.*v*. with GA (0.2 mg/kg) and the accumulation of **3** in the bile at 4 h was 12-, 1.5 × 10^3^-, 32-, and 15-fold those of GA, 3MGA, **1**, and **2**, respectively (Fig. 3E). We confirmed that **3** was the major metabolite of GA eliminated into the bile in SD rats. GA, **3**, and **2** were found in the feces of SD rats collected for 24 h after the *i*.*v*. injection of GA (0.2 mg/kg), while 3MGA and **1** were not found in the feces (Fig. 3F).

### Transport of compounds 2 and 3 into tubular cells

The pooled plasma samples collected from EHBRs 12 h after oral treatment with GA (200 mg/kg) were loaded onto ultracentrifuge filters with a 1 × 10^4^ molecular weight cut-off and concentrated by centrifugation. The concentrations of **2**, **3**, and GA in the pooled plasma were 7.8, 11.5 µM, and 7.0 µM, respectively. **2**, **3**, and GA in the filtrate were undetectable (based on the detection limits, they were at levels less than 3.2 nM for **2** and **3**, less than 16 nM for GA) by LC-MS/MS analysis. The albumin-binding ratios of **2** and **3** in the plasma were calculated to be more than 99.9% and that of GA was more than 99.7%.

**Figure 4:**
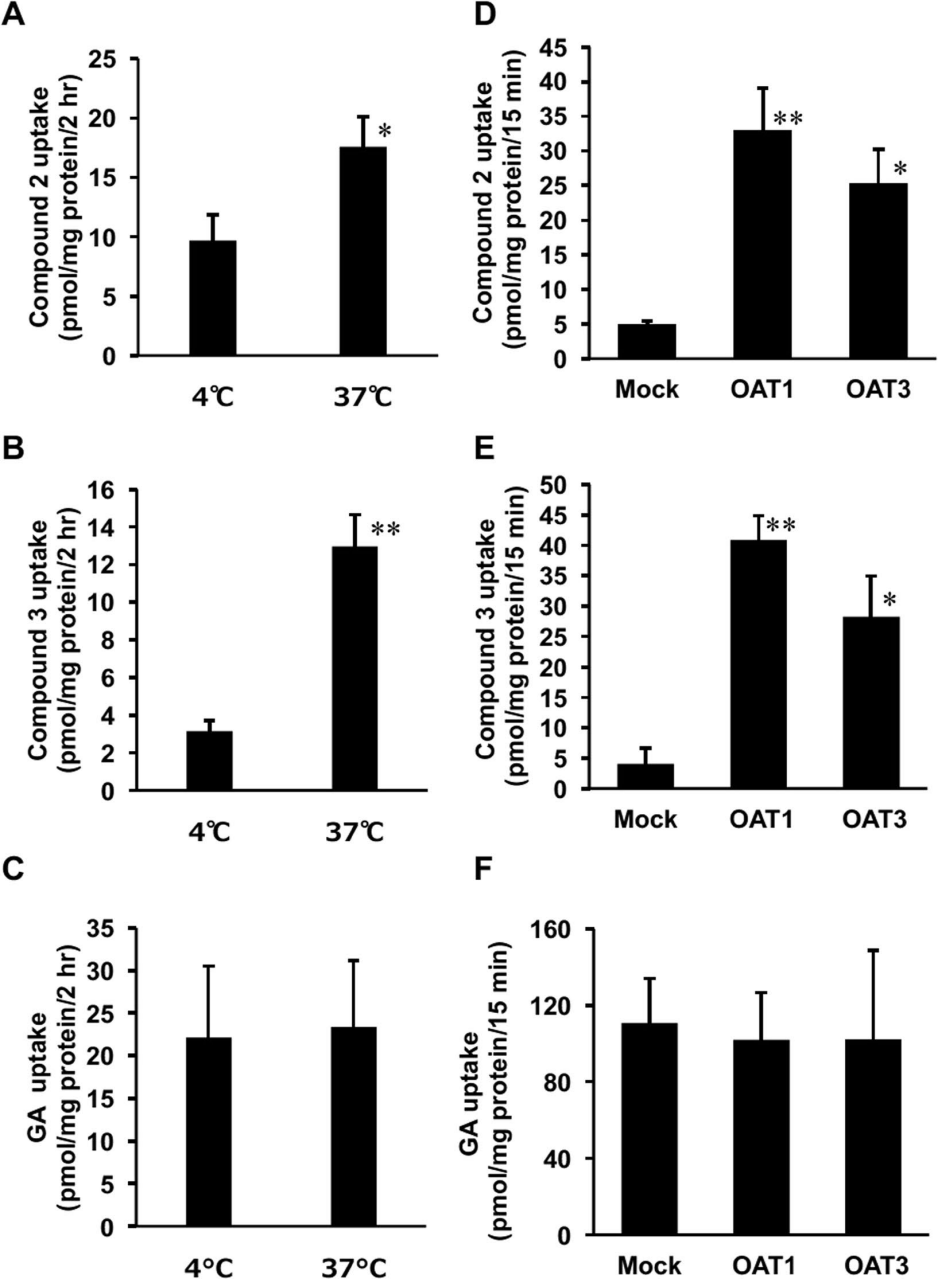
Uptake of **2** and **3** into rat kidney slices (**A**–**C**) or into MDCK II cells stably expressing OAT1 or OAT3 (**D**–**F**). Female EHBRs were orally treated with GA (200 mg/kg), and their plasma samples were collected 24 h after the treatment. The concentrations of compounds **2**, **3**, and GA in the plasma samples of EHBRs were 7.8, 11.5, and 7.0 µM, respectively. Kidneys were collected from normal SD rats, and the kidney slices were incubated with the plasma samples of EHBRs at 37 °C or 4 °C for 2 h (**A–C**). MDCK II cells stably expressing OAT1 or OAT3 were incubated with the plasma samples of EHBRs at 37 °C for 15 min (**D**–**F**). Then, the concentrations of compounds **2**, **3**, and GA in kidney slices or the cells were measured by LC-MS/MS. Data are expressed as mean ± S.E. (*n* = 3 for (**C, D**), and (**E**); *n* = 6 for (**A, B**), and F). *P < 0.05 and **P < 0.01 *vs* 4 °C group by Student’s *t*-test for (**A–C**), and *vs* mock cells by Dunnett’s multiple *t*-test for (**D–F**).

Kidney slices were prepared from SD rats and incubated with the pooled plasma, the pH of which pH was adjusted to 5.5 at 4 °C or 37 °C for 2 h. Figure 4A–C show the amounts of **2**, **3**, and GA accumulated in the kidney slices. The uptakes of **2** and **3** by the kidney slices incubated at 37 °C were significantly higher (*p* < 0.05 and 0.01, respectively) than those incubated at 4 °C, although the uptakes of GA were not different in the samples incubated at 4 °C or 37 °C.

MDCK II cells stably expressing OAT1, 3, or mock cells were incubated with pooled plasma collected from EHBRs, the pH of which was adjusted to 5.5 at 37 °C for 15 min, and the uptake of **2**, **3**, and GA into the cells was measured (Fig. 4D–F). Although the uptake of GA into the cells expressing OAT1 or 3 was approximately the same as that into mock cells, the uptake of compounds **2** and **3** into the cells expressing OAT1 or 3 was significantly higher (*p* < 0.01 and 0.05, respectively) than that into mock cells.

### Concentrations of GL metabolites in the plasma of a patient with pseudoaldosteronism

The patient was a 76-year-old female who had taken a Kampo formula containing licorice for 3 years (1.5 g of licorice for 1 year and 2 months, and 3 g for 1 year and 10 months). On day 0, we found a low plasma potassium level (2.1 mEq/l) and a high creatinine kinase level (364 U/l). Since low plasma renin activity and a low aldosterone level were also found, the patient was diagnosed with pseudoaldosteronism due to licorice. The administration of the Kampo formula was stopped and potassium supplementation of up to 100 mEq/day was started. In the plasma collected on day 0, we detected compound **3** at 8.6 µM, GA at 1.3 µM, and **2** at 87 nM, while **1**, GL and 3MGA were not detected. On day 5, the plasma potassium level was still 2.1 mEq/l and potassium supplementation was continued. However, the plasma concentrations of **3** and GA had decreased to 3.6 µM and 0.65 µM, respectively. **2**, **1**, GL, and 3MGA were not detected. On day 13, the plasma potassium level was increased to 2.8 mEq/l, and the concentrations of **3** and GA were 61 nM and 11 nM, respectively. On day 14, the plasma potassium level was 3.4 mEq/l, and the concentration of **3** was 57 nM; GA was not detected. On day 18, the plasma potassium level had recovered to a normal level (4.9 mEq/l), so potassium supplementation was stopped. On this day, the concentration of **3** was below the detectable limit.

### Dot-blot analysis and ELISA of GL metabolites using anti-3MGA-mAb

**Figure 5:**
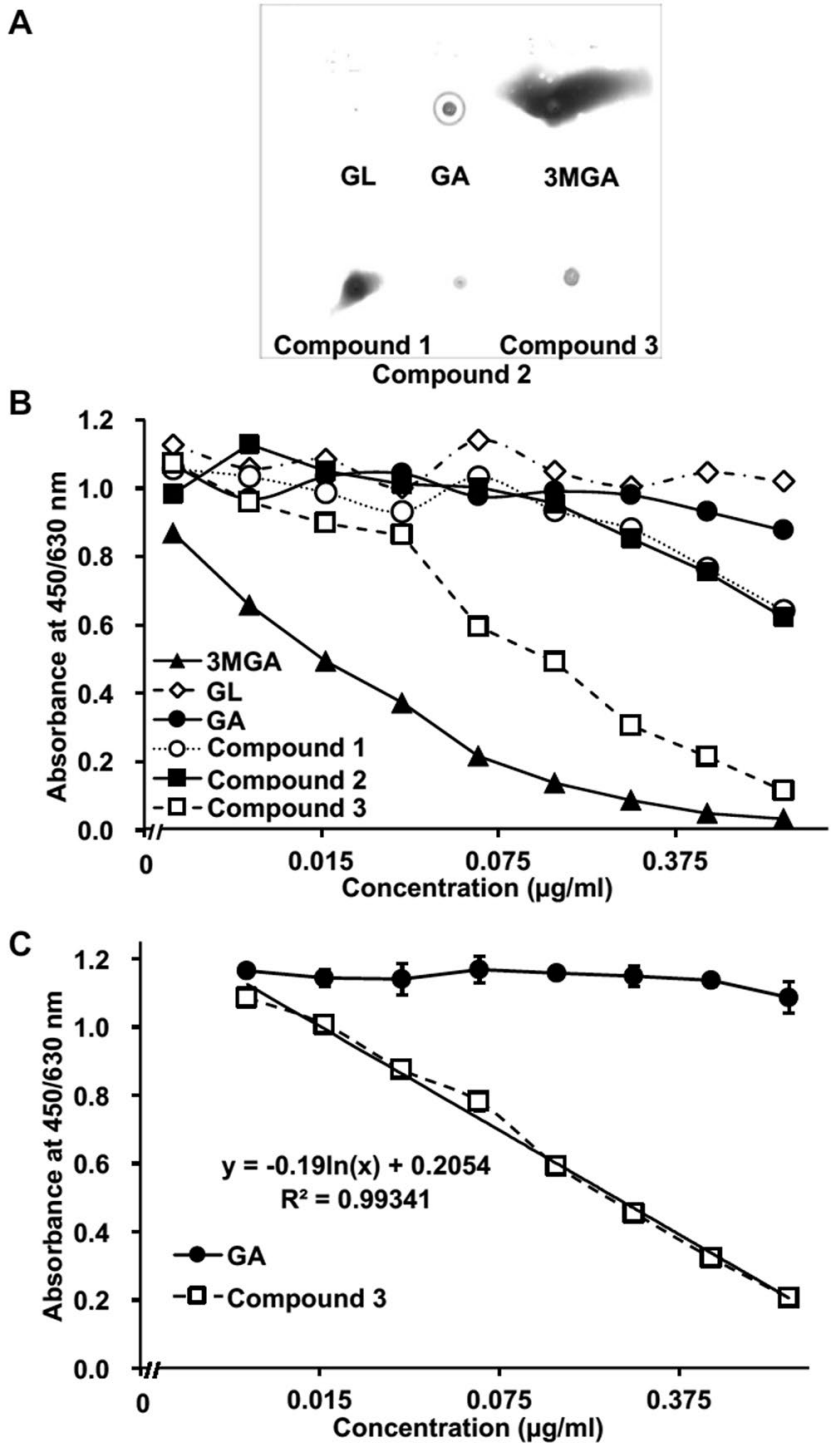
Dot-blot analysis and ELISA detection range of GL metabolites using anti-3MGA-monoclonal antibody (mAb). GL metabolites (1 µg each) were spotted on a PES membrane, and colored using an anti-3MGA-mAb (**A**). Competitive ELISA using anti-3MGA-mAb for GL metabolites was performed. Data are represented from a single analysis (*n* = 1) (**B**). Competitive ELISA using an anti-3MGA-mAb for compound **3** and GA was performed. Data are represented as mean ± S.D. (*n* = 3). Two-way ANOVA indicated a significant difference between GA and **3** (F1,36 = 4441, *P* < 0.001), concentrations (F8,36 = 315, *P* < 0.001), and their interactions (F8,36 = 281, *P* < 0.001). The calibrated line for the absorbance-concentration profile of **3** was calculated by the linear least-squares method, and the regression formula is shown in graph (**C**).

We confirmed the cross-reactivity of anti-3MGA-mAb against these GL metabolites. GL, GA, 3MGA, and compounds **1**, **2**, and **3** (1 µg each) were spotted on a polyethersulfone (PES) membrane, fixed onto the membrane, and colored using an anti-3MGA-mAb (Fig. 5A). By imaging analysis, the areas of positive staining (in pixels) were follows: GL, not detectable; GA, 97; 3MGA, 3395; **1**, 606; **2**, 79; and **3**, 146. Thus, the anti-3MGA-mAb cross-reacted to some extent with other metabolites of GL.

Figure 5B shows the concentration profiles of GL metabolites and absorbance in a competitive ELISA system by using anti-3MGA-mAb. When the specificity of the mAb to 3MGA was calibrated to 100%, the cross-reactivity for GL, GA, **1**, **2**, and **3** was 0.23%, 2.2%, 4.8%, 4.0%, and 23%, respectively. As mentioned above, **3** is suggested as the causative agent of pseudoaldosteronism in humans, where the plasma of patients taking licorice mainly contains both **3** and GA. We confirmed the selectivity of the anti-3MGA-mAb in discriminating between **3** and GA and established the detectable measurement range for **3** in this ELISA system. Two-way ANOVA revealed that the anti-3MGA-mAb had significant selectivity for **3** compared with GA (*P* < 0.001), and the detectable concentration range for **3** was 7.8 ng/ml (14 nM) to 1.0 µg/ml (1.8 µM) (Fig. 5C).

## Discussion

In the present study, we isolated a new GA metabolite **2** along with **3** in the urine of Mrp2-deficient EHBRs treated with GA. Since **2** and **3** were detected in both the plasma and the urine of EHBRs, but not in those of SD rats treated with GA, and since these metabolites were observed in the bile of SD rats, **2** and **3** must be substrates for Mrp2. When EHBRs were orally treated with GA, an increase in plasma GA was observed first, followed by an increase in **3**, then in **2**, and finally in **1**. The concentrations of GA, **1**, **2**, and **3** were approximately 10 µM 12 h after oral treatment with GA. This suggests that the order of GA metabolism would be 3-*O*-sulfate conjugation by sulfotransferase, 22-hydroxylation by CYP, and then 30-glucuronic acid conjugation by glucuronyltransferase in the liver. Under normal conditions, **3** may soon be eliminated from the liver into the bile *via* Mrp2, where the concentrations of **2** and **1** in the bile of SD rats injected *i*.*v*. with GA were much lower than that of **3**. Since we could detect **3** in the bile and GA in the feces of SD rats treated *i*.*v*. with GA, **3** would be hydrolyzed into GA by the enteric bacteria and reabsorbed into the circulation *via* enterohepatic circulation. This was supported by the biphasic profile of plasma GA concentration in SD rats orally treated with GA. Finally, GA would be eliminated into the feces as GA or **3**. On the other hand, the concentrations of 3MGA in the plasma and the urine of EHBRs orally treated with GA were much lower than those of **1**, **2**, and **3**, revealing that 3MGA is a minor metabolite of GA in EHBRs.

We found that most of **2** and **3** in plasma was bound to albumin, suggesting that these two compounds would not be eliminated into the urine *via* glomerular filtration, but rather *via* tubular secretion. As **1** had a high binding ratio to albumin and was a substrate for OAT1 and 3^18^ and **2** and **3** share a high binding ratio to albumin, these compounds can be transported into the cells actively *via* OAT1 and 3, and eliminated into the urine by tubular secretion. As GA also has a high binding ratio to albumin but is not a substrate for OATs, GA was not detected in the urine in either SD rats or EHBRs. The IC_50_ values of GL and its metabolites on 11*β*-HSD2 using a rat kidney microsome fraction were as follows: **3** (0.10 µM),**2** (0.11 µM), 3MGA (0.25 µM)^15^, GA (0.31 µM)^15^, GL (1.8 µM)^15^, and **1** (2.0 µM)^18^. However, the present results suggest that **1**, **2**, and **3**, rather than GA, can inhibit 11*β*-HSD2 to cause pseudoaldosteronism *in vivo* because 11*β*-HSD2 is existed in the microsomes of tubular cells^16^.

EHBRs treated with GA eliminated GA metabolites through the urine, with **2** accumulating to the highest level, followed by **3**, **1**, and then 3MGA. Since the plasma concentrations of **1**, **2**, and **3** 12 h after oral treatment with GA were all approximately 10 µM (Fig. 3B), these differences observed in the urine may reflect to the affinities of these metabolites for the transporters expressed on the membrane of tubular epithelial cells. However, we could not analyze their affinities to OAT1 and 3 since the amounts of these compounds isolated from the urine of EHBRs were not sufficient for the uptake experiments. Further studies are needed to explain why the major metabolite of GA in the urine of EHBRs treated with GA is **2**.

In the present study, we analyzed a plasma sample collected from a patient who regularly ingested licorice as a part of a Kampo formula and suffered from pseudoaldosteronism, in which we detected primarily **3**. Although Kato *et al*. detected 3MGA in the blood of the patients suffering from pseudoaldosteronism^10^, the concentration of 3MGA in the plasma sample in the present study was below the detection limit (less than 3.2 nM). The analytical method in the Kato’s study was reversed-phase HPLC with UV detection at 254 nm^9^. Since we could not achieve a good separation of 3MGA and **3** using reversed phase octadecyl silica gel (ODS) column, we employed a Scherzo SM-C18 column (Imtakt, Kyoto, Japan), which is a multi-mode ODS column with anion and cation exchange. This column provided good separation of **1**, **2**, **3**, 3MGA, GA, and GL. In our unpublished data, we analyzed more than 30 plasma samples collected from the patients who consumed licorice and suffered from pseudoaldosteronism. The major metabolites of GL in these plasma samples were GA and **3**, and 3MGA was not detected in any of the samples (data not shown). With regard to the relationships between the symptoms of pseudoaldosteronism and the plasma concentrations of GA and **3** in the patients, we will report on this in a subsequent article. It is possible that Kato *et al*. could assign the peak of **3** in the HPLC chromatogram of human plasma sample to 3MGA.

In a previous study, we discovered **1** from pooled urine of EHBRs guided by a positive signal in eastern blot analysis using an anti-3MGA-mAb^18^. However, we could not detect 3MGA, **2**, or **3** in the urine of EHBRs by eastern blot analysis, although the concentrations of **2** and **3** were substantially higher than those of **1** and 3MGA. When equal amounts of **1**, **2**, and **3** were spotted onto the PES membrane and stained using anti-3MGA-mAb, the positive signals of **2** and **3** were much less than that of compound **1** (Fig. 5A). However, when we confirmed the cross-reactivities of the anti-3MGA-mAb with these GA metabolites, higher affinity was observed for **3** followed by that of 3MGA. In general, the adsorption of small molecular compounds onto the membrane is low. We used 1-ethyl-3-(3′-dimethylaminopropyl) carbodiimide hydrochloride (EDC) and *N*-hydroxysuccinimide (NHS) as the activator of the carboxyl group, especially the glucuronyl motif, to bind BSA and adhere to the PES membrane as a hapten-carrier protein conjugate. 3MGA and **1** have the glucuronyl motif and may be fixed more efficiently onto the membrane, whereas there are no glucuronyl motifs in the structures of **2** and **3**. Although the anti-3MGA-mAb has high affinity for **3** as determined by competitive ELISA, **3** might be removed from the PES membrane by the washing process in the eastern or dot-blot analysis, preventing the acquisition of a strong signal. On the other hand, it may be possible to detect **3** in the plasma or the urine of patients with sufficient sensitivity through competitive ELISA using anti-3MGA-mAb.

In conclusion, the present study revealed that the major metabolites of GA in EHBRs were **1**, **2**, and **3**, rather than 3MGA, and that these metabolites might be candidates for the causative agents of licorice-induced pseudoaldosteronism. We detected **3** as the major metabolite of GL in the plasma of patients suffering from licorice-induced pseudoaldosteronism instead of 3MGA. **3** can be detected in plasma or urine using anti-3MGA-mAb and can be used as a marker compound to prevent pseudoaldosteronism when licorice is prescribed to patients. The analysis of these GL metabolites in the plasma and urine of patients is ongoing and clinical results will be reported in the future.

## Methods

### Chemicals, reagents, animals, and general procedures

All chemicals, reagents, animals, and general procedures were the same as those described in our previous study^18^. The animal experimental procedures were approved by the Animal Care Committee at the Graduate School of Pharmaceutical Sciences, Nagoya City University, Nagoya, in accordance with the guidelines of the Japanese Council on Animal Care.

### Isolation of compounds 2 and 3

Mrp2-deficient female EHBRs (6-week-old, Japan SLC, Hamamatsu, Japan) were reared with drinking water containing 1 mg/ml GA suspended in 1% carboxymethylcellulose (CMC) solution for 3 months, and their urine was collected and pooled. One liter of pooled urine (dried weight, approximately 40 g) was evaporated under reduced pressure after filtration. The concentrated urine (400 ml) was partitioned with EtOAc and *n*-BuOH. The *n*-BuOH-soluble materials (4.2 g) were subjected to ODS silica gel column chromatography (MeOH/H_2_O/trifluoro acetic acid (TFA) 0:100:0.1 → 100:0:0.1). A fraction eluted with MeOH/H_2_O/TFA (60:40:0.1) was further separated by silica gel column chromatography (CHCl_3_/MeOH/H_2_O/TFA 1:0:0:0 → 6:4:1:0.01), from which a fraction eluted with CHCl_3_/MeOH/TFA (9.1:0.9:0.01 and 8:2:0.01) was further purified by C_18_ HPLC (Cosmosil 5C_18_-ARII (Nacalai Tesque, Kyoto, Japan), 5 µm, 10 mm *i*.*d*. × 250 mm, solvent MeCN/H_2_O/TFA, 29:71:0.1, flow rate 2.5 ml/min, detection 254 nm) to afford **2** (1.9 mg). A fraction eluted with MeOH/H_2_O/TFA (80:20:0.1) in the ODS silica gel column chromatography was further separated by silica gel column chromatography (CHCl_3_/MeOH/TFA 1:0:0 → 0:1:0.01), from which a fraction eluted with CHCl_3_/MeOH/TFA (9.5:0.5:0.01 and 9.2:0.8:0.01) was further purified by C_18_ HPLC (Cosmosil 5C_18_-ARII, 5 µm, 10 mm *i*.*d*. × 250 mm, solvent MeCN/H_2_O/TFA, 45:55:0.1, flow rate 2.5 ml/min, detection 254 nm) to afford **3** (0.4 mg).

Compound **2**: colorless amorphous solid; [α]_D_^21^ +47 (*c* 1.0, MeOH); UV (MeOH) *λ*_max_ 249 (*ε* 6289) nm; ECD (MeOH) *λ* (*Δε*) 229 (+8.3) nm; ^1^H-NMR (CD_3_OD, 500 MHz) and ^13^C-NMR (CD_3_OD, 125 MHz), see Table 1; ESIMS *m/z* 565 [M-H]^−^; HRESIMS *m/z* 565.2835 [M-H]^−^ (calcd for C_30_H_45_O_8_S, 565.2835).

18*β*-glycyrrhetyl-3-*O*-sulfate (**3**): [α]_D_^21^ +103 (c 0.2, MeOH); ECD (MeOH) *λ* (*Δε*) 230 (+7.7) nm; ^1^H-NMR (CD_3_OD, 500 MHz) *δ* 5.58 (1H, s, H-12), 3.95 (1H, dd 12.0, 4.5 Hz, H-3), 2.73 (1H, brd 13.5 Hz, H-1a), 2.48 (1H, s, H-9), 2.20 (1H, brd 14.5 Hz, H-18), 2.14 (1H, dd 14.0, 4.5 Hz, H-16a), 2.07 (1H, m, H-2a), 1.94 (1H, m, H-21a), 1.87 (1H, m, H-15a), 1.85 (1H, m, H-19a), 1.81 (1H, m, H-2b), 1.75 (1H, m, H-7a), 1.72 (1H, m, H-19b), 1.65 (1H,brd 13.0 Hz, H-6a), 1.60 (1H, m, H-6b), 1.45 (1H, m, H-7b), 1.44 (3H, s, H-27), 1.41 (1H, m, H-21b), 1.40 (2H, m, H-22), 1.25 (1H, brd 12.5 Hz, H-15b), 1.17 (3H, s, H-29), 1.16 (3H, s, H-25), 1.15 (3H, s, H-26), 1.06 (3H, s, H-23), 1.05 (1H, m, H-1b), 1.04 (1H, m, H-16b), 0.87 (1H, m, H-5), 0.86 (3H, s, H-24), 0.84 (3H, s, H-28) and ^13^C-NMR (CD_3_OD, 125 MHz) *δ* 202.6 (C-11), 180.5 (C-30), 172.9 (C-13), 128.9 (H-12), 87.2 (C-3), 63.0 (C-9), 56.6 (C-5), 49.9 (C-18), 46.7 (C-8), 44.9 (C-20), 44.6 (C-14), 42.4 (C-19), 40.1 (C-1), 39.9 (C-4), 39.0 (C-22), 38.2 (C-10), 33.8 (C-7), 33.0 (C-17), 32.0 (C-21), 29.2 (C-28), 28.7 (C-23), 28.7 (C-29), 27.6 (C-15), 27.4 (C-16), 25.2 (C-2), 23.8 (C-27), 19.3 (C-26), 18.6 (C-6), 17.0 (C-25), 16.9 (C-24); ESIMS *m/z* 549 [M-H]^-^; HRESIMS *m/z* 549.2888 [M-H]^-^ (calcd for C_30_H_45_O_7_S, 549.2886).

### Determination of *in vitro* 11*β*-HSD2 activity using rat kidney microsomes

Assays were conducted as described by Diederich *et al*.^20^ with slight modifications as described in our previous report^15^.

### Pharmacokinetic experiments of GA metabolites in SD rats or EHBRs orally treated with GA

Female SD rats or EHBRs (9-week-old) were anesthetized by an intraperitoneal (*i*.*p*.) injection of urethane (1 g/kg) and their jugular veins were exposed. GA suspended in 0.5% CMC was then administered orally (200 mg/kg) to the unconscious rats, and blood samples were collected from the jugular vein; urine samples were collected using a metabolic cage at an appropriate time over a 12-h period. In another experiment, GA (0.2 mg/kg) in DMSO was injected into a vein of female SD rats (8-week-old), and their feces were collected for 24 h using a metabolic cage. Feces were homogenized in phosphate-buffered saline (PBS; 0.15 M, pH 7.2), centrifuged (1.2 × 10^4^ *g*, 10 min), and the supernatants were collected. After 1 week as a washout period, female SD rats were anesthetized by an *i*.*p*. injection of urethane (1 g/kg) and their biliary tract was cannulated. GA (0.2 mg/kg) was injected into the jugular vein, and the bile was collected every hour for 4 h. Aliquots (10 µl) of plasma, urine, homogenate of feces, or bile were mixed with 20 µl of subtilisin (0.91 U/ml) and incubated at 37 °C for 30 min, followed by the addition of 70 µl of astragaloside IV solution (1 µg/ml in ethanol containing 0.5% formic acid, used as internal standard), and kept at −20 °C for 30 min. After centrifugation (2 × 10^4^ *g* for 7 min), the concentrations of compounds **1**, **2**, **3**, 3MGA, GA, and GL in the supernatant of the samples prepared from plasma and urine were measured using LC-ESIMS/MS under the following conditions: column, Scherzo SM-C18 (3 μm, 3 mm *i*.*d*. × 100 mm); mobile phase, (A) 5 mM AcNH_4_, (B) 125 mM AcNH_4_/MeCN 1:4, at a flow rate of 0.3 ml/min, with the following gradient profile: A:B = 50:50–0:100 (0–3 min) and 0:100 (3–10 min). The transitions (precursor to daughter) monitored and retention times were as follows: ESI(+) 743.4 to 567.5 *m/z* for compound **1** (5.5 min), ESI(−) 565.5 to 96.5 *m/z* for **2** (6.2 min), ESI(−) 549.5 to 96.5 *m/z* for **3** (8.1 min), ESI(+) 647.6 to 453.6 *m/z* for 3MGA (9.2 min), ESI(+) 471.3 to 91.0 *m/z* for GA (8.8 min), ESI(+) 823.5 to 453.6 *m/z* for GL (10.8 min), and ESI(+) 785.4 to 143.0 *m/z* for astragaloside IV (3.0 min). Linear regressions over the concentration range of 32 nM to 20 µM for each compound were examined using the peak-area ratio of the compounds to their internal standards and the least-squares method (*r*^2^ > 0.98).

### Uptake of compounds 2 and 3 by rat kidney slices and cells expressing OAT1 and 3

Uptake studies using rat kidney slices and cells stably expressing OAT1 and OAT3 were conducted with the same protocol as in our previous study^18^. We used the same pooled plasma of female EHBRs orally treated with GA for 12 h as in our previous study^18^.

### Assay of binding of compounds 2 and 3 to serum albumin

We measured the binding ratios of compounds **2** and **3** to serum albumin by the same protocol using pooled plasma of female EHBRs as in our previous study^18^.

### Patient suffering from licorice-induced pseudoaldosteronism

The patient was a 76-year-old female. She provided written informed consent to the use of her blood and urine samples for this study, which was approved by the Independent Ethics Committee of Kameda General Hospital, in accordance with the Declaration of Helsinki. Her anamnesis was follows: hypertension, systemic lupus erythematosus, and antiphospholipid antibody syndrome (for 15 years), necrotic pulmonary aspergillosis (6 years ago), cerebral infarction (14 and 4 years ago, two times), and stomach cancer (3 years ago). After the operation for stomach cancer, the patient had been treated with Kracie Juzentaihoto Extract Granules for Ethical Use (Kracie Pharmaceuticals., Tokyo, Japan). The daily dosage of this product includes 6.2 g of a dried extract of the following mixed crude drugs: 3.0 g of the dried root of *Astragalus propinquus*, 3.0 g of the dried bark of *Cinnamomum cassia*, 3.0 g of the dried root of *Rehmannia glutinosa*, 3.0 g of the dried root of *Paeonia lactiflora*, 3.0 g of the dried rhizome of *Cnidium officinale*, 3.0 g of the dried rhizome of *Atractylodes japonica*, 3.0 g of the dried root of *Angelica acutiloba*, 3.0 g of the dried root of *Panax ginseng*, 3.0 g of the dried sclerotium of *Wolfiporia cocos*, and 1.5 g of the dried root and stolon of *Glycyrrhiza uralensis*. All crude drugs and Juzentaihoto Extract were standardized by Japanese Pharmacopoeia 17th Edition^21^. A tendency for low plasma potassium (around 3 mEq/l) subsequently appeared, and the patient was supplemented with potassium gluconate (40 mEq/day). Other concurrent medications were prednisolone (2.5 mg every 2 days) and voriconazole (200 mg/day), etc. The patient had complained of malaise, weakness, and difficulty of walking and was found to have an extremely low plasma potassium level and a high creatinine kinase level.

### Dot-blot analysis using anti-3MGA-mAb

Anti-3MGA-mAb and 3MGA-human serum albumin (HSA)-conjugate were developed by the same protocols established in our previous studies^22,23^. GL, GA, 3MGA, **1**, **2**, and **3** (1 µg each) were spotted on a Mustang^TM^ E positively charged PES membrane (Pall Co., East Hills, NY, USA). The membrane was then immersed into 0.1 M MES buffer (pH 4.7) containing 2% EDC and 1% NHS, and kept at room temperature for 1 h to activate the carboxyl group. After washing with distilled water, PBS containing 1% bovine serum albumin (BSA) was added to the membrane, which was shaken at room temperature for 2 h for fixation. Next, the membrane was blocked with PBS containing 5% skim milk (Morinaga-milk, Tokyo, Japan) and 0.1% Tween 20 at 4 °C overnight to reduce nonspecific adsorption. After washing twice with PBS containing 0.1% Tween 20 (T-PBS) for 5 min, the membrane was incubated with anti-3MGA-mAb and kept at room temperature for 3 h with constant agitation. The membrane was then washed twice with T-PBS for 5 min and treated with 1:500 diluted horseradish peroxidase (HRP)-labeled goat anti-mouse IgG (whole molecule; Sigma, St. Louis, MO, USA) antibody in T-PBS for 1 h with gentle shaking. Finally, the membrane was washed twice with T-PBS and once with PBS, and exposed to 1 mg/ml of freshly prepared 4-chloro-1-naphthol-0.03% H_2_O_2_ in PBS for 15 min at room temperature. The reaction was stopped by washing with distilled water, and the immunostained PES membrane was allowed to dry and then photographed. The positively stained areas were measured using Image J 1.46r (http://imageJ.nih.gov/ij).

### ELISA system using anti-3MGA-mAb

3MGA-HSA (1 µg/ml in 50 mM carbonate buffer, pH 9.6) was added into a 96-well plate (Nagel Nunc, Penfield, NY, USA) (100 µl in a well) and incubated at 37 °C for 1 h. After the removal of 3MGA-HSA, the wells were washed three times with T-PBS (200 µl in a well). The wells were incubated with 300 µl in a well of 5% skim milk at 4 °C overnight. After the wells had been washed with T-PBS three times, the sample solution diluted with Can Get Signal^®^ Solution 1 (Toyobo Co., Ltd., Osaka, Japan) (50 µl in a well) and anti-3MGA-mAb (1:4,000 dilution with Can Get Signal^®^ Solution 1) (50 µl in a well) were added to the wells, and the plate was incubated at 37 °C for 1 h. After the wells had been washed with T-PBS three times, HRP-labeled goat anti-mouse IgG (Fc specific) antibody (Sigma, 1:20,000 dilution with Can Get Signal^®^ Solution 2) was added into the wells (100 µl in a well) and the plate was incubated at 37 °C for 1 h. Next, the wells were washed with T-PBS three times and the substrate solution (3,3′,5,5′-tetramethylbensidine, TMB solution; Fujifilm Wako Pure Chemical Corporation, Osaka, Japan) was added to the wells (100 µl in a well), followed by incubation at room temperature for 5 min. Then, the stop solution (0.5 M sulfuric acid) was added to the wells (100 µl in a well) and the optical density was measured at 450 nm/630 nm.

### Statistics

Statistical analysis for the groups treated with compounds **2** and **3** as shown in Fig. 2 and the groups shown in Fig. 4D–F was performed using one-way analysis of variance (ANOVA) and Dunnett’s multiple *t*-test by PASW Statistics (version 18, SPSS; IBM, Armonk, NY, USA). Statistical analysis for the groups treated with GA in Fig. 2 and the groups in Fig. 4A–C was performed using Student’s *t*-test in Microsoft Excel^®^. Statistical analysis for comparison of the concentration-absorbance profiles of the groups as shown in Fig. 5C was performed by two-way ANOVA using R (https://www.r-project.org/). Calibrated line and the regression formula in Fig. 5C were calculated by the linear least-squares method using Microsoft Excel^®^. A probability value of less than 0.05 was considered to indicate statistical significance.
